# The Relationship Between Clinicopathological Features and Prognosis of 22 Cases of Tubular Breast Carcinoma

**DOI:** 10.1155/tbj/5599018

**Published:** 2025-02-03

**Authors:** Lin Tian, Xiangchao Meng, Huiyan Si, Yue Qiu, Rui Qu, Hongye Chen

**Affiliations:** ^1^Department of Thyroid and Breast Surgery, The First Peoples Hospital of Zunyi/Third Affiliated Hospital of Zunyi, Zunyi 563000, Guizhou, China; ^2^Department of General Surgery, The First Medical Center of Chinese PLA General Hospital, Beijing 100853, China; ^3^Department of Breast Surgery, Qinhuangdao First Hospital, Qinhuangdao 066000, Hebei, China

**Keywords:** breast, clinicopathological features, molecular types, prognosis, tubular carcinoma

## Abstract

**Background:** Breast tubular carcinoma is a special pathological type of invasive breast cancer, accounting for about 0.8% to 10.0% of breast cancer cases, and it is a rare type of breast cancer. Currently, there is still a lack of relevant diagnostic and treatment consensus. Exploring the relationship between the pathological characteristics, molecular subtypes, and prognosis of ductal breast cancer is of great scientific value and clinical significance for improving patients' survival rate and quality of life.

**Methods:** The clinical data of 22 patients with tubular breast carcinoma diagnosed by pathology in The First Medical Center of PLA General Hospital from January 2001 to December 2021 were collected, and their pathological features, molecular classification, and prognosis were analyzed retrospectively.

**Results:** The clinicopathological features of 22 patients with tubular breast carcinoma were age ≥ 35 years, married, tumor ≤ 2 cm, single focal, mixed type, no lymph node metastasis, estrogen receptor (ER) positive, progesterone receptor (PR) positive, Ki-67 ≤ 14%, CyclinD1 negative, less recurrence, and metastasis. Twenty-two patients with breast tubular carcinoma were followed up for 5 years after surgery, and the survival rate of disease-free survival (DFS) was 90.9% (20/22). The positive rates of ER, PR, and human epidermal growth factor receptor-2 (HER-2) are 100.0%, 100.0%, and 40.9%, respectively. The proportion of tumor cells expressing Ki-67 is 45.4%. Among them, the difference of HER-2 level, recurrence and metastasis, and postoperative comprehensive treatment showed different prognoses.

**Conclusion:** Tubular breast carcinoma is a kind of tumor with a low malignant degree. The prognosis is significantly related to its HER-2 level, recurrence and metastasis, and postoperative comprehensive treatment by univariate analysis, in which HER-2 is an independent risk factor, postoperative comprehensive treatment is a protective factor, but postoperative recurrence and metastasis have nothing to do with the prognosis by the multivariate analysis.

## 1. Introduction

According to the classification of breast tumors stipulated by WHO (5th Edition) [1, 2], tubular breast cancer can be divided into pure type and mixed type. Pure tubular carcinoma is defined as the proportion of a tubule structure > 90% [3]. Mixed tubule carcinoma is diagnosed when the tubule composition accounts for 50%–90%, accompanied by other types of cancer meanwhile, usually intraductal carcinoma [3]. Studies have showed that tubular breast cancer, accounting for 0.8%–10% of invasive breast cancer, has low malignancy and good prognosis [4, 5]. The research into tubular breast cancer contributes to our understanding and recognition of this disease, increasing the accuracy and timeliness of diagnoses. Through studying tubular breast cancer, we have the potential to identify more effective treatments and strategies. This not only increases survival rates for patients but also enhances their quality of life. The research into tubular breast cancer could offer new insights into the ways cancer grows and spreads, providing a deeper understanding that may be beneficial for other types of cancer as well. In this study, clinical data of tubular breast cancer patients were retrospectively analyzed to explore their clinicopathologic features, molecular typing, and prognosis, in order to provide evidence for individualized clinical diagnosis and treatment.

## 2. Materials and Methods

### 2.1. General Information

The clinical data of 22 patients with breast tubular carcinoma in The First Medical Center of PLA General Hospital from January 2001 to December 2021 were collected, and the general data (age, marital, menstrual state, tumor size, and multifocal), pathological features, molecular classification, and prognosis of the patients were recorded.

### 2.2. Pathological Features

All the puncture and surgical biopsy specimens for pathological diagnosis were fixed with 10% formalin and sectioned after paraffin embedding, followed by routine HE staining for histomorphology and immunohistochemical analysis. The diagnostic criteria for ductal breast carcinoma are based on the WHO classification of breast cancer histopathology, which relies on the comprehensive diagnosis of morphological features such as uniform cell size, well-defined borders, growth pattern, less cellular pleomorphism, limited necrosis, and mitotic figures. The expressions of estrogen receptor (ER), progesterone receptor (PR), human epidermal growth factor receptor-2 (HER-2), Ki-67, and CyclinD1 were detected by the SP method. According to the intensity and integrity of cell membrane staining, HER-2 immunohistochemical staining was divided into (−), (+), (++), and (+++). HER-2 positive was defined as immunohistochemical staining (+++) or positive FISH detection. Ki-67 ≥ 15% was positive, ≤ 14% was negative, and ≥ 30% was highly expressed.

### 2.3. Follow-Up Visit

Follow-up was conducted by telephone and outpatient review. The median follow-up time was 93 months until August 2022. The follow-up time was defined as the time from the date of treatment to the death of the patient or the follow-up deadline. Overall survival was defined as the time from the date of treatment to the patient's death or last follow-up. Disease-free survival (DFS) was defined as the time from the date of treatment to clinical or imaging evidence indicating ipsilateral breast or regional lymph nodes and distant metastasis.

### 2.4. Statistical Analysis

IBM SPSS 25.0 software (SPSS, Inc., Chicago, IL, USA) was used for statistical analysis. *χ*^2^ test and Fisher's exact test were used to compare the categorical variables. Multivariate logistic regression was used for the multivariate analysis. The difference was statistically significant (*p* < 0.05).

## 3. Results

### 3.1. General Situation

All the 22 patients with breast tubular carcinoma were female, ranging from 29 to 83 years old, with the mean age of 50.82 years, and the median age of 48 years, including one case of < 35 years and 21 cases of ≥ 35 years. Twenty-two cases were married. There were 14 cases before menopause and eight cases after menopause. Tumor ≤ 2 cm in 18 cases, > 2 cm in four cases, and multifocal and multicenter in three cases, all with two lesions. Axillary lymph node metastasis occurred in two cases, and the number of metastatic lymph nodes was one and 4four, respectively, as shown in Table 1.

### 3.2. Pathological Features

Among the 22 breast tubular carcinoma patients, there were eight cases of pure tubular carcinoma and 14 cases of mixed-type tubular carcinoma. The most common associated pathological type was intraductal carcinoma. The pathologic types associated with mixed tubular carcinoma included intraductal carcinoma (*n* = 8), invasive ductal carcinoma (*n* = 5), invasive ethmoid carcinoma (*n* = 5), invasive lobular carcinoma (*n* = 3), and invasive ethmoid carcinoma + ductal carcinoma in situ (*n* = 1). One case was accompanied with invasive ethmoid carcinoma and ductal carcinoma in situ, as shown in Table 1.

### 3.3. Molecular Typing

**Figure 1:**
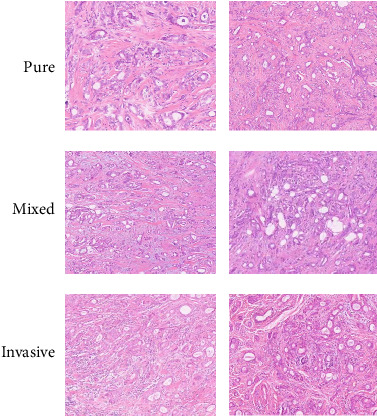
Representative immunohistochemical images including pure, mixed, and invasive breast tubular carcinoma.

Immunohistochemical detection was performed in all 22 patients, and representative images are shown in Figure 1. Both ER and PR were positive in all cases (100.0%). Nine cases (40.9%) were HER-2 positive. The Ki-67 test was performed in all 22 cases, and the Ki-67 positivity rate was defined as > 14% of tumor cells expressing the marker. Of the 22 patients, 10 (45.4%) showed Ki-67 positivity. For the pure tubular carcinoma type, 8 cases were Ki-67-positive, and for the mixed-type carcinoma, the Ki-67 positivity rate was 63.6% (9/14). CyclinD1 was detected in 22 cases, with five cases (22.7%) showing positivity. The distribution of these molecular markers is shown in Table 1.

### 3.4. Clinical Treatment

Among the 22 patients, 59.1% (13/22) underwent mastectomy, 36.4% (8/22) underwent breast-conserving surgery, and 4.5% (1/22) underwent interventional microwave ablation. Axillary lymph node treatment: Sentinel lymph node biopsy (SLNB) rate was 45.5% (10/22), axillary lymph node dissection rate was 50% (11/22), and axillary lymph node puncture biopsy was negative and untreated 4.5% (1/22). The complete rate of comprehensive therapy (chemotherapy, radiotherapy, endocrine therapy, or targeted therapy) was 95.5% (21/22), recurrence rate 4.5% (1/22), metastasis rate 4.5% (1/22), and 5-year survival rate 90.9% (20/22), as shown in Table 1.

### 3.5. Follow-Up and Prognosis

A total of 20 out of 22 patients with breast tubular carcinoma were followed up for 5 years after surgery. The 5-year DFS rate was 90.9% (20/22), meaning 20 patients did not experience recurrence during the follow-up period. There were no significant differences in age, marital status, menstrual status, tumor size, multifocal or multicenter tumors, axillary lymph node metastasis, the number of metastatic lymph nodes, ER, PR, Ki-67 expression, type of breast surgery, or axillary treatment (*χ*^2^ test, *p* > 0.05). However, statistically significant differences were observed in the recurrence and metastasis rates (*p* < 0.05), as shown in Table 2.

### 3.6. Multivariate Analysis of Prognosis

Multiple logistic regression analysis and Exp (B) odds ratio OR analysis were performed for 20 of the 22 patients up to 5 years after surgery. HER-2 level was an independent risk factor, postoperative comprehensive treatment was a protective factor, and postoperative recurrence and metastasis had nothing to do with the prognosis, as shown in Table 3.

## 4. Discussion

Breast tubular carcinoma is a special pathological type of invasive breast cancer, accounting for about 0.8%–10.0% of breast cancer [3]. It was first described by Carnil in 1869, and McDivitt first named tubular carcinoma in 1968 [6]. Breast tubular carcinoma has no obvious particularity in clinical manifestation, and it is not easy to distinguish from other types of breast cancer in ultrasound, molybdenum target, and nuclear magnetic resonance examination [7, 8]. However, tubular carcinoma of the breast has good biological behavior, similar characteristics, and better survival rate compared with low-grade ductal carcinoma of the breast [7, 8]. This indicates the necessity and adequacy of histopathological examination and reinforces the designation of tubular carcinoma as a distinct subtype of breast cancer.

In this study, we retrospectively analyzed the clinicopathological features, molecular subtypes, and prognosis of 22 patients with tubular breast carcinoma. The results showed that tubular breast carcinoma mainly affects middle-aged and elderly women, and the tumors tend to be small in size with rare lymph node metastasis. Both pure and mixed subtypes were identified, with intraductal carcinoma being the most common pathological type associated with the mixed subtype. Immunohistochemically, tubular breast carcinoma typically exhibits positive ER and PR expression and low Ki-67 proliferation. HER-2 positivity was found in one case only. The univariate analysis identified HER-2 status, recurrence/metastasis, and postoperative adjuvant therapy as prognostic factors. The multivariate analysis further validated HER-2 overexpression as an independent risk factor and adjuvant treatment as a protective prognostic factor. Postoperative recurrence/metastasis did not significantly impact the prognosis. This cohort study confirms the favorable characteristics and excellent prognosis typically associated with tubular breast carcinoma. The malignancy is low, with longer DFS and extremely low recurrence/metastasis rates. Standard surgical treatment along with individualized adjuvant therapy based on risk stratification can achieve satisfactory clinical outcomes. Larger prospective studies are still needed to validate the prognostic relevance of clinicopathological and molecular features in tubular breast carcinoma.

In conclusion, tubular breast carcinoma represents a distinctive subtype of invasive breast cancer with indolent behavior and good response to standard treatment approaches. Further elucidating its biological phenotype may improve personalized management strategies for this patient population. Zhang et al. included 68 patients with a median follow-up time of 64.3 months. Previous reports have showed low rates of local recurrence and distant metastasis, as well as high survival rates for pure ductal carcinoma and mixed ductal carcinoma [9]. The results of this study showed that the patients were mainly middle-aged and elderly; the tumors were small, multifocal, and multicenter; and axillary lymph node metastasis was rare, which was basically consistent with previous research results. However, mixed tubular carcinoma was more common in this group, and mainly associated with intraductal carcinoma, while other invasive carcinoma was relatively rare, and invasive ethmoid carcinoma was an invasive carcinoma with a good prognosis. Some scholars found that the life expectancy of patients with pure type was similar to that of healthy women in the same age by comparing patients with a pure type of breast tubular carcinoma and Stage I breast ductal carcinoma [10–12]. The results of this study showed that there was no significant difference in the prognosis between mixed and pure type, that may be related to intraductal carcinoma, the main complication of mixed tubular carcinoma, which needs further study.

Axillary lymph node metastasis and tumor size are important factors affecting the prognosis of breast cancer, while tubular carcinoma is characterized by smaller tumor, lower tissue grade, and lower axillary lymph node metastasis rate [13, 14]. Livi et al. included 307 patients with a median follow-up time of 8.4 years, and the recurrence rate was 1.9% to 4.7%, believed that the prognosis of simple tubular carcinoma is better than that of mixed tubular carcinoma, and the prognosis of patients without lymph node metastasis is better than that of lymph node positive cases [15]. However, some studies have suggested that lymph node positivity does not affect the disease-free or overall survival of tubular carcinoma patients, and there is still a fairly good prognosis, because when tubular carcinoma metastases to lymph nodes, the number is usually small, rarely more than three, and most of them are located at Level I [13, 16, 17]. Zandrino et al. proposed that the tumor volume of breast tubular carcinoma has nothing to do with the prognosis, which is the special difference between it and other breast cancers [18]. Our study also showed that the prognosis of breast tubular carcinoma was not significantly related to tumor size, presence or absence of axillary lymph node metastasis, and the number of metastasis.

Immunohistochemical results of 20 cases of breast tubule carcinoma showed that ER and PR were almost positive, Ki-67 index was low, and HER-2 expression was negative. The high ER and PR positivity in tubular carcinoma has been consistently noted in the literature, with our study showing 100% positivity for both markers. This finding underscores the hormone-sensitive nature, making endocrine therapy a cornerstone of the treatment. ER/PR positivity in breast cancer typically suggests a low-grade, hormone-responsive tumor, which is associated with a more favorable prognosis. The Ki-67 proliferation index is widely used as a marker of tumor aggressiveness. In our study, the Ki-67 positivity rate was 45.4%, with a higher rate in mixed-type tubular carcinoma (63.6%) compared to the pure-type (8/22). These findings are consistent with the previous research, suggesting that the mixed type may exhibit slightly higher proliferative activity compared to the pure type, potentially indicating a more aggressive biological behavior in some cases. Due to the lack of myoepithelialsic components, myoepithelial markers p63 and SMA were negative. CyclinD1 is a weak oncogene that has been amplified or enhanced in a variety of tumors. In this study, CyclinD1 was detected in six cases with a positive rate of 83.3% (5/6). Min et al. showed that the positive rate of ER, PR, and HER-2 overexpression was 92.9%, 87.0%, and 12.9% in breast tubular carcinoma [19]. The positive rate of HER-2 in breast tubular carcinoma is low, and lymph node metastasis is less frequent [13, 20]. This study showed that the prognosis of patients with high and low expression of HER-2 was significantly different (*p* < 0.05), and it was an independent prognostic factor of breast tubular carcinoma. Of note, tubular carcinoma is generally considered to be negative or low expression of HER-2 protein. The higher-than-expected 40.9% HER-2-positive rate may come from the more heterogeneous tumor, potentially including areas of more aggressive cancer that could be HER-2-positive. Although most tubular carcinomas are low-grade and HER-2-negative, there may be some biological variation in rare cases, leading to HER-2 positivity. These cases are less typical but may occur, especially in larger or more aggressive tumors, or due to changes in tumor biology over time.

At present, conventional treatment is commonly used in the treatment of breast tubular carcinoma in China, while foreign reports suggest that breast conserving surgery can also achieve satisfactory therapeutic effects [21]. Rakha et al. believe that breast cancer has low invasiveness and good prognosis, and breast conserving surgery may be the best treatment, especially for patients without lymph node metastasis [22]. The postoperative comprehensive treatment may be a protective factor, and the impact of specific treatments (e.g., chemotherapy, endocrine therapy, and radiotherapy) on patient outcomes remains to be studied, which would allow a more precise assessment of their individual or combined effects, thereby aiding clinicians in crafting personalized treatment plans. Some large retrospective studies and meta-analyses have also put forward the view that the recurrence risk of breast tubular carcinoma is low, and recurrence and metastasis are not easy to predict by tumor size or node status, and axillary lymph node metastasis is not common in this disease [8, 23]. Some researchers have also suggested that the risk of axillary lymph node involvement in pure tubular carcinoma of the breast is very low [24, 25], and SLNB can be omitted for patients with focus < 1 cm [24], but others emphasized that surgical axillary evaluation is warranted even in such cases [25]. Admittedly, the good prognosis for breast tubular carcinoma is associated with its lower rate of lymph node metastasis. However, lymph node metastasis is sometimes found even in patients with small tumors (≤ 1 cm) [26]. Therefore, axillary lymph node staging should still be considered in all patients with tubular carcinoma of the breast [19, 27]. Among the 22 patients with breast tubular carcinoma in this study, 59.1% (13/22) underwent mastectomy, 36.4% (8/22) underwent breast conserving surgery, 4.5% (1/22) underwent interventional microwave ablation, 45.5% (10/22) underwent SLNB, 50% (11/22) underwent axillary lymph node dissection, and 4.5% (1/22) of axillary lymph nodes were biopsy negative without treatment. The results showed that there was no significant difference in the prognosis among different methods of breast operation and different management of axillary lymph nodes. The combination of ER/PR positivity, low Ki-67, and the generally low HER-2 expression suggests that endocrine therapy should be the backbone of treatment, with the possibility of adjuvant chemotherapy for high-risk patients. However, the low recurrence rate and excellent prognosis seen in this patient cohort indicate that many patients can be managed effectively with breast-conserving surgery followed by adjuvant endocrine therapy, without the need for more aggressive chemotherapy regimens.

Breast tubular carcinoma is a special histological subtype of invasive ductal carcinoma, with pathological characteristics including the following [28, 29]: Unique morphological features: the cells of ductal breast carcinoma exhibit a tendency to form tubular or glandular structures; uniform cell size: these cancer cells typically have a uniform size distribution, with generally low nuclear grade, indicating less activity of the disease; clear borders: in addition, ductal carcinoma tumors usually have well-defined borders; growth pattern: the cells of ductal breast carcinoma tend to grow in an angular, infiltrative, cord-like, and tubular fashion; limited cellular pleomorphism: compared to other types of breast cancer, ductal carcinoma cells demonstrate less cellular variation; limited necrosis and mitotic figures: these are also features that can help distinguish ductal carcinoma from other cancers. For patients who are ER and/or PR negative, treatment can be based on the conventional histopathological classification of breast cancer after redoing immunohistochemical diagnosis. In pN0M0TC patients undergoing breast-conserving surgery, postoperative radiotherapy is a favorable prognostic factor, indicating that adjuvant radiotherapy should be considered as a standard treatment for these patients [30–32]. In this study, the rate of comprehensive treatment (endocrine therapy, chemotherapy, targeted therapy, and radiotherapy) was 95.5%, the 5-year survival rate was 85.7%, the recurrence rate was 4.5%, and the metastasis rate was 4.5%. The statistical results showed that there was a significant difference in the prognosis with or without postoperative adjuvant therapy (*p* < 0.05), and it was an independent protective factor for the prognosis. However, there was no significant relationship between recurrence, metastasis, and prognosis. In this study, the completion rate of comprehensive therapy (endocrine therapy, chemotherapy, targeted therapy, and radiotherapy) was 95.5%, 5-year survival rate was 85.7%, recurrence rate was 4.5%, and metastasis rate was 4.5%. There is also limitation of small sample size in detecting statistically significant associations and limited follow-up duration, and larger multicenter studies are needed to validate these findings. Also, histological grade and vascular invasion would enhance the depth of the pathological analysis. The necessity and outcomes of chemotherapy and radiotherapy for low-risk patients could be explored. Potential biases in patient selection and treatment variability may also affect the results. Although the study identifies HER-2 positivity and postoperative comprehensive treatment as prognostic factors, how these factors influence outcomes across different molecular subtypes or surgical approaches is known. Modifications to current guidelines on axillary surgery and adjuvant therapy, particularly in light of the favorable prognosis and low incidence of lymph node metastasis in tubular carcinoma are suggested.

To sum up, breast tubular carcinoma has lower incidence, better histological grade, earlier clinical and pathological stage, lower lymph node metastasis rate, good immunohistochemical results, longer DFS, extremely low local recurrence and metastasis rate, and good prognosis. It is a kind of special invasive breast cancer with a low malignant degree. Breast-conserving surgery and standard management of axillary lymph nodes are the development direction of the current surgical methods, supplemented by postoperative radiotherapy and individual endocrine therapy and/or chemotherapy can achieve satisfactory results.

## Figures and Tables

**Table 1:** Clinical and pathological features of 22 patients with breast tubular carcinoma.

Clinicopathological features	*n*	%
*Age (years)(n = 22)*		
< 35	1	4.5
≥ 35	21	95.5

*Marital status (n = 22)*		
Unmarried	0	0
Married	22	100

*Menstrual state (n = 22)*		
Premenopausal period	14	63.6
Postmenopausal	8	36.4

*Tumor size (cm)(n = 22)*		
≤ 2	18	81.8
> 2	4	18.2

*Multifocal and multicenter(n = 22)*		
No	19	86.4
Yes	3	13.6

*Pathological types (n = 22)*		
Pure type	8	36.4
Mixed type	14	63.6

*Coexistence of mixed types*		
Intraductal carcinoma	8	36.4
Invasive carcinoma	14	63.6

*Axillary lymph node metastasis (n = 22)*		
No	20	90.9
Yes	2	9.1

*Number of lymph node metastasis*		
≤ 3	20	90.9
≥ 4	2	9.1

*ER status (n = 22)*		
Negative	0	0
Positive	22	100.0

*PR status (n = 22)*		
Negative	0	0
Positive	22	100.0

*HER-2 status (n = 22)*		
Negative	13	59.1
Positive	9	40.9

*Ki-67 status (n = 22)*		
≤ 14%	12	54.5
≥ 15%, < 30%	9	40.9
≥ 30%	1	4.5

*CyclinD1 status (n = 22)*		
Negative	17	77.3
Positive	5	22.7

*Breast operation method (n = 22)*		
Breast conserving surgery	8	36.4
Mastectomy	13	59.1
Interventional microwave ablation	1	4.5

*Axillary lymph node treatment (n = 22)*		
Sentinel lymph node biopsy	11	50.0
Axillary lymph node dissection	11	50.0

*Comprehensive treatment (n = 22)*		
Yes	14	63.6
No	8	36.4

*Recurrence and metastasis*		
Recurrence	1	4.5
Metastasis	1	4.5
None	20	90.9

*Five years of survival (n = 22)*		
Yes	20	90.9
No	2	9.1

Abbreviations: ER = estrogen receptor; PR = progesterone receptor.

**Table 2:** Univariate analysis of the prognosis in 22 patients with breast tubular carcinoma.

Variable	5 years DFS rate (%)	*χ* ^2^	*p*
Age (years)		0.105	0.746
< 35	1/1		
≥ 35	19/21		
Marital status		—	—
Unmarried	—		
Married	20/22		
Menstrual state		0.177	0.674
Premenopausal period	13/14		
Postmenopausal period	7/8		
Tumor size (cm)		9.90	0.002
≤ 2	18/18		
> 2	2/4		
Multifocal and multicenter		0.347	0.556
No	17/19		
Yes	3/3		
Pathological types		0.177	0.674
Pure type	7/8		
Mixed type	13/14		
Coexistence of mixed types		1.257	0.262
Intraductal carcinoma	8/8		
Invasive carcinoma	12/14		
Axillary lymph node metastasis		0.220	0.639
No	18/20		
Yes	2/2		
Number of lymph node metastasis		0.220	0.639
0	18/20		
> 0	2/2		
ER status		—	—
Negative			
Positive	20/22		
PR status		—	—
Negative			
Positive	20/22		
HER-2 status		0.075	0.784
Negative	12/13		
Positive	8/9		
Ki67 status		0.153	0.926
≤ 14%	11/12		
≥ 15%, < 30%	8/9		
≥ 3 0%	1/1		
CyclinD1 status		0.647	0.421
Negative	15/17		
Positive	5/5		
Breast operation method		0.243	0.885
Breast conserving surgery	7/8		
Mastectomy	12/13		
Interventional microwave ablation	1/1		
Axillary lymph node treatment		0.000	1.000
Sentinel lymph node biopsy	9/10		
Axillary lymph node dissection	10/11		
Comprehensive treatment		0.177	0.674
Yes	13/14		
No	7/8		
Recurrence and metastasis		22.000	< 0.001
Recurrence	0/1		
Metastasis	0/1		
None	20/20		
Molecular typing		0.335	0.563
Luminal A	6/7		
Luminal B	14/15		

Abbreviations: DFS = disease-free survival; ER = estrogen receptor; HER-2 = human epidermal growth factor receptor 2; PR = progesterone receptor.

**Table 3:** Multivariate prognostic analysis of 22 patients with breast tubule carcinoma.

5 years DFS a	*B*	Standard error	Wald	Degree of freedom	Significance	Exp (B)
Intercept	424.12	0.000	0.07	1	0.94	29.49
HER-2 level	46.62	0.000	0.01	1	0.99	0.65
Comprehensive treatment	54.60	0.000	−0.01	1	0.99	−0.59
Recurrence and metastasis	211.65		−0.09	0	0.93	−19.45

Abbreviations: DFS = disease-free survival; HER-2 = human epidermal growth factor receptor 2.

## Data Availability

The data that support the findings of this study are available on request from the corresponding authors.

## References

[1] Lebeau A. (2021). Updated WHO Classification of Tumors of the Breast. *Pathologe, Der*.

[2] Lebeau A., Denkert C. (2021). Updated WHO Classification of Tumors of the Breast: The Most Important Changes. *Pathologe, Der*.

[3] Tan P. H., Ellis I., Allison K. (2020). The 2019 World Health Organization Classification of Tumours of the Breast. *Histopathology*.

[4] Izquierdo M., Tresserra F., Rodriguez I. (2012). 167 the Clinical Features and Prognosis of Tubular Breast Cancer. *European Journal of Cancer*.

[5] Izquierdo Sanz M., Tresserra Casas F., Nacho Rodríguez G. (2011). Clinical Features and Prognosis of Tubular Breast Cancer. *Breast Cancer Research*.

[6] Stalsberg H., Hartmann W. H. (2000). The Delimitation of Tubular Carcinoma of the Breast. *Human Pathology*.

[7] Fritz P., Bendrat K., Sonnenberg M. (2014). Tubular Breast Cancer. A Retrospective Study. *Anticancer Research*.

[8] Metovic J., Bragoni A., Osella-Abate S. (2021). Clinical Relevance of Tubular Breast Carcinoma: Large Retrospective Study and Meta-Analysis. *Frontiers in Oncology*.

[9] Zhang W. W., Wu S. G., Ling Y. H. (2018). Clinicopathologic Characteristics and Clinical Outcomes of Pure Type and Mixed Type of Tubular Carcinoma of the Breast: A Single-Institution Cohort Study. *Cancer Management and Research*.

[10] Sakhri S., Bouheni M., Jaidane O., Addouni O., Chargui R., Rahal K. (2020). Pure Tubular Carcinoma of the Breast: A Rare Entity. *International Journal of Gynecological Cancer*.

[11] Poirier E., Desbiens C., Poirier B. (2018). Characteristics and Long-Term Survival of Patients Diagnosed With Pure Tubular Carcinoma of the Breast. *Journal of Surgical Oncology*.

[12] Fedko M., Scow J., Shah S. (2010). Pure Tubular Carcinoma of the Breast. *Annals of Surgical Oncology*.

[13] Saridakis A., Berger E., Reisenbichler E. (2021). Tubular Carcinoma of the Breast: Patterns of Incidence, Axillary Lymph Node Metastasis and Survival. *Annals of Surgical Oncology*.

[14] Sun J. Y., Zhou J., Zhang W. W., Li F. Y., He Z. Y., Wu S. G. (2018). Tubular Carcinomas of the Breast: An Epidemiologic Study. *Future Oncology*.

[15] Livi L., Paiar F., Meldolesi E. (2005). Tubular Carcinoma of the Breast: Outcome and Loco-Regional Recurrence in 307 Patients. *European Journal of Surgical Oncology*.

[16] Chen S. L., Zhang W. W., Wang J., Sun J. Y., Wu S. G., He Z. Y. (2019). The Role of Axillary Lymph Node Dissection in Tubular Carcinoma of the Breast: A Population Database Study. *Medical Science Monitor*.

[17] Ueo T., Di C. T. (2014). Could Sentinel Lymph Node Biopsy Be Omitted in Tubular Carcinoma of the Breast?-A Retrospective Single Centre Study. *Apmis*.

[18] Zandrino F., Calabrese M., Faedda C., Musante F. (2006). Tubular Carcinoma of the Breast: Pathological, Clinical, and Ultrasonographic Findings. A Review of the Literature. *La radiologia medica*.

[19] Min Y., Bae S. Y., Lee H. C. (2013). Tubular Carcinoma of the Breast: Clinicopathologic Features and Survival Outcome Compared with Ductal Carcinoma In Situ. *Journal of Breast Cancer*.

[20] Oakley G. J., Tubbs R. R., Crowe J. (2006). HER-2 Amplification in Tubular Carcinoma of the Breast. *American Journal of Clinical Pathology*.

[21] Perkins G. H., Middleton L. P., Tran R. T., Garcia S. M., Buchholz T. A. (2005). Tubular Carcinoma: Outcomes Analysis of Favorable Breast Cancer Treated with Breast Conserving Therapy. *Breast Cancer Research and Treatment*.

[22] Rakha E. A., Lee A. H. S., Evans A. J. (2010). Tubular Carcinoma of the Breast: Further Evidence to Support Its Excellent Prognosis. *Journal of Clinical Oncology*.

[23] Lea V., Gluch L., Kennedy C. W., Carmalt H., Gillett D. (2015). Tubular Carcinoma of the Breast: Axillary Involvement and Prognostic Factors. *ANZ Journal of Surgery*.

[24] Kara H., Arikan A. E., Dulgeroglu O., Tokat F., Uras C. (2021). Pure Tubular Carcinoma of the Breast: Is Axillary Staging Necessary?. *Indian Journal of Surgery*.

[25] Dejode M., Sagan C., Campion L. (2013). Pure Tubular Carcinoma of the Breast and Sentinel Lymph Node Biopsy: A Retrospective Multi-Institutional Study of 234 Cases. *European Journal of Surgical Oncology*.

[26] Stolnicu S., Moldovan C., Resetkova E. (2016). Even Small Pure Tubular Carcinoma of the Breast (Stage T1a and T1b) Can Be Associated with Lymph Node Metastases-the U T MD Anderson Cancer Center Experience. *European Journal of Surgical Oncology*.

[27] Ramzi S., Hyett E. L., Wheal A. S., Cant P. J. (2018). The Case for the Omission of Axillary Staging in Invasive Breast Carcinoma that Exhibits a Predominant Tubular Growth Pattern on Preoperative Biopsy. *Breast Journal*.

[28] Wilson B. E., Jacob S., Barton M. B. (2019). Global Demands for Chemotherapy, HER-2 and Endocrine Therapy for Breast Cancer Using NCCN Resource Stratified Guidelines. *Asia-Pacific Journal of Clinical Oncology*.

[29] Gradishar W. J., Moran M. S., Abraham J. (2021). Breast Cancer, Version 4.2021 Featured Updates to the NCCN Guidelines. *Journal of the National Comprehensive Cancer Network*.

[30] Zhao Y. T., Chai N., Li S. Y. (2023). Evaluation of the Efficacy of Chemotherapy for Tubular Carcinoma of the Breast: A Surveillance, Epidemiology, and End Results Cohort Study. *Cancer Medicine*.

[31] Romano A. M., Wages N. A., Smolkin M., Fortune K. L., Atkins K., Dillon P. M. (2015). Tubular Carcinoma of the Breast: Institutional and SEER Database Analysis Supporting a Unique Classification. *Breast Disease*.

[32] Stauber J., Chevli N., Haque W. (2021). Prognostic Impact of Radiation Therapy in Tubular Carcinoma of the Breast. *Radiotherapy & Oncology*.

